# Partially Bonded Crystals: A Pathway to Porosity and Polymorphism

**DOI:** 10.1021/acsnano.4c06489

**Published:** 2025-01-28

**Authors:** Carina Karner, Emanuela Bianchi

**Affiliations:** † Institut für Theoretische Physik, TU Wien, Wiedner Hauptstraße 8-10, A-1040 Wien, Austria; ‡ CNR-ISC, Uos Sapienza, Piazzale A. Moro 2, 00185 Roma, Italy

**Keywords:** patchy colloids, self-assembly, shape-anisotropy, frustration, polymorphism

## Abstract

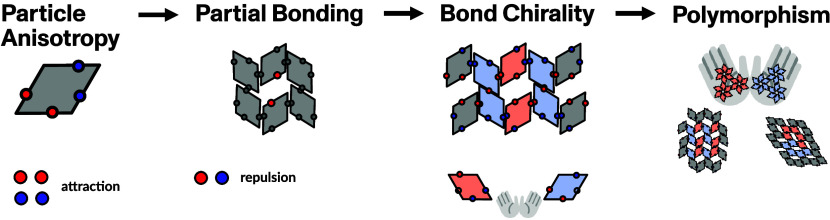

In recent years, experimental and theoretical investigations have shown that anisotropic colloids can self-organize into ordered porous monolayers, where the interplay of localized bonding sites, so-called patches, with the particle’s shape is responsible for driving the systems away from close-packing and toward porosity. Until now it has been assumed that patchy particles have to be fully bonded with their neighboring particles for crystals to form, and that, if full bonding cannot be achieved due to the choice of patch placement, disordered assemblies will form instead. In contrast, we show that by deliberately displacing the patches such that full bonding is disfavored, a different route to porous crystalline monolayers emerges, where geometric frustration and partial bonding are decisive process. The resulting dangling bonds lead to the emergence of effectively chiral units which then act as building blocks for energetically equivalent crystal polymorphs.

With recent advancements in colloidal synthesis techniques it has become possible to fabricate colloidal particles with precise control over their size, shape and interaction profiles.^1−6^ Via self-assembly, these nano- to micron-sized building blocks can be used for bottom-up materials design, where properties of the self-assembled material can be engineered by fine-tuning the particle characteristics. A particularly relevant subclass of colloidal particles in this context are patchy particles, where only specific sections of the particle’s surface are functionalized.^1,2,7−23^ This selective functionalization allows the particles to bond exclusively at these “patchy” regions, making it possible to exploit the intuitive connection between the patch bonding pattern and the target crystal symmetry.

In experiments, it has been shown that the assembly of spherical units is largely controlled by the location and size of the patches, as demonstrated by polystyrene particles with hydrophobic gold patches on both poles.^12^ These so-called triblock particles assemble into a porous Kagome lattice as long as the size of the polar patches allows two bonds per patch. When this condition is satisfied, bonding is maximized in a triangular local geometry, leading to the extended Kagome monolayer. Similar patchy design principles have been successfully exploited to assemble the highly desired colloidal diamond from particles with tetragonal patch symmetry.^11,24^ For small patches, the one bond per patch limit applies and the intuitive relationship between patch number, size and placement on the particle surface becomes even more apparent. As small patches on spherical units are still hard to achieve in experiments, one has to rely also on simulations. These show that one patch particles typically assemble dimers,^14,17^ two patch systems assemble finite clusters or chains,^10,18,19^ while extended structures have only been observed starting from three patches,^20^ where the symmetry of the patch arrangement directly impacts the symmetry of the assembly. Examples are two-dimensional assemblies, where spherical particles with four patches placed along the sphere perimeter at 90° intervals assemble into a square lattice, or where particles with six evenly distributed patches yield hexagonal crystals.^20,21^ With the same rationale even dodecagonal quasicrystals can be assembled by placing five patches equally spaced along the particle perimeter, as this creates a local neighborhood with the 5-fold symmetry required for the growth of quasicrystals.^22,23^

Besides number and size of patches, changing the particle shape from spherical to aspherical adds another dimension for parameter exploration. When the shape of the particles is anisotropic, some mutual particle orientations might be favored with respect to others on a purely entropic basis. The interplay between shape and patchiness creates a delicate balance between entropic and enthalpic factors opening up more possibilities for intricate particle arrangements. Examples of assemblies resulting from such a delicate balance are colloidal crystals of nonspherical colloidal platelets with functionalized edges or localized ligands, such as nanocrystals, DNA origami and polymer-based particles.^25−34^

These lego-like design principles are also being exploited for inverse design, where given the target structure, the building block shape, and the patch size and position are determined by using Machine Learning algorithms.^35−40^

Ultimately, be it by intuiting how patchy particles bond locally or by employing optimizing strategies, the underlying assumption remains–by design–that the desired crystalline or quasi-crystalline target structures result from all patches being bonded to at least one other patch. This raises a central question: what happens to the self-assembly of patchy particles if they are prevented from fully bonding due to conflicting interactions?

Here, we focus on a class of patchy platelets with the shape of regular rhombi and four localized bonding sites (one per edge) of two types: while patches of the same type attract each other, patches of opposite type repel each other. Additionally, bond selectivity is tuned by considering different arrangements of the two patch types along the platelet perimeter. Extensive investigations have shown that these patchy rhombi tend to assemble into fully bonded, close-packed tilings as long as their patches are placed in the center of their edges, irrespective of their bond selectivity pattern.^29,41,42^ In contrast, when the patches are placed off-center, a complex interplay sets in, where the pairwise alignment resulting from the particle shape opposes the enthalpic drive to maximize the bonding, often leading to off-edge bonding between the particles.^29^ The formation of off-edge bonds opens up the possibility for porous assemblies that can be either fully^29,41^ or partially^42^ bonded, depending on the interplay between patch position and bond selectivity. While fully bonded open assemblies usually result in crystalline porous monolayers, where porosity is controlled by the degree of off-edge bonding between the particles,^29,41^ partially bonded assemblies lead to the formation of extensive disordered networks.^42^ Even though the emergence of these disordered networks seems to be a robust scenario in all patchy rhombi systems where geometric bond frustration is present,^29,42^ the emergence of non-fully bonded crystals cannot be in principle ruled out.

## I Results

**Figure 1:**
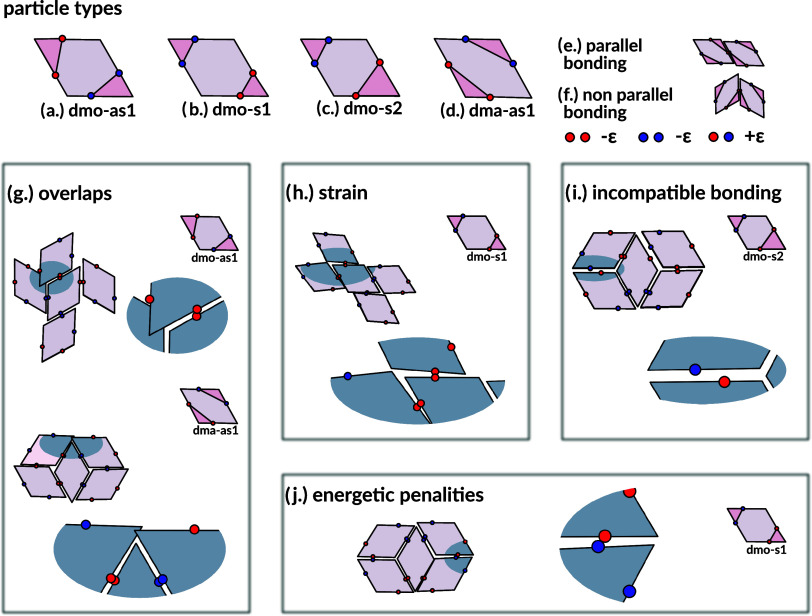
Particle types, pair bonding motifs and full bonding failures. All studied particle types (top row) and example bonding motifs where full bonding either fails or is disfavored (framed panels). To facilitate the distinction between the particle types, we highlight the triangle area spanned by patches of the same type, with their enclosed edge in a darker shade. The particle types are (a) dmo-as1 (b) dmo-s1, (c) dmo-s2, (d) dma-as1. In (e, f) we depict the two modes of bonding available for rhombus particles: parallel (e) and nonparallel (f). In (g) we show motifs with overlaps for dmo-as1 and dma-as1; (h) depicts a dmo-s1 motif where full bonding is possible only under strain; (i) shows an instance for incompatible bonding for dmo-s2, where relative patch placement prevents full bonding; in (j) we depict how the bonding motif can force patches of different type to face each other, resulting in an energetic penalty, as exemplified by dmo-s1.

Leveraging on the described framework and on our experience, we consider a subset of four arbitrary, but carefully selected, particle types, namely dmo-as1, dmo-s1, dmo-s2 and dma-as1, as shown in Figure 1a–d. The particle naming scheme leans on previous nomenclature from ref (29) and is specified in the Section III, where details of the model features and parameters are reported. Despite the displacement of the patches with respect to their central position being relatively small (as it amounts to 10% of the length of the rhombi edge), the patch arrangement and bond selectivity, together, strongly disfavor or even prevent the formation of fully bonded assemblies. In particular, the investigated systems have been selected to introduce geometric bond frustration via at least one of the full bonding failures depicted in Figure 1g–j (for a more exhaustive set of failing bonding motifs see ref (42)): in the selected systems, the four patches of a particle cannot simultaneously bond to the neighboring particles without incurring either overlaps, incompatible bonding (due to steric hindrance), energetic penalties (due to patches repelling each other), or at least a certain degree of strain induced by patches bonding almost out of interaction range. In order to robustly assess the effect of geometric bond frustration on self-assembly, we conduct large scale real-space Monte Carlo simulations, performing a substantial total of 24,576 separate simulations–considering all 16 parallel runs for each of the 384 state points across all 4 particle topologies–lasting from 2 to 2.5 × 10^7^ Monte Carlo sweeps (see the Section III for more details).

### I.I Bonding Properties at the Particle Level

**Figure 2:**
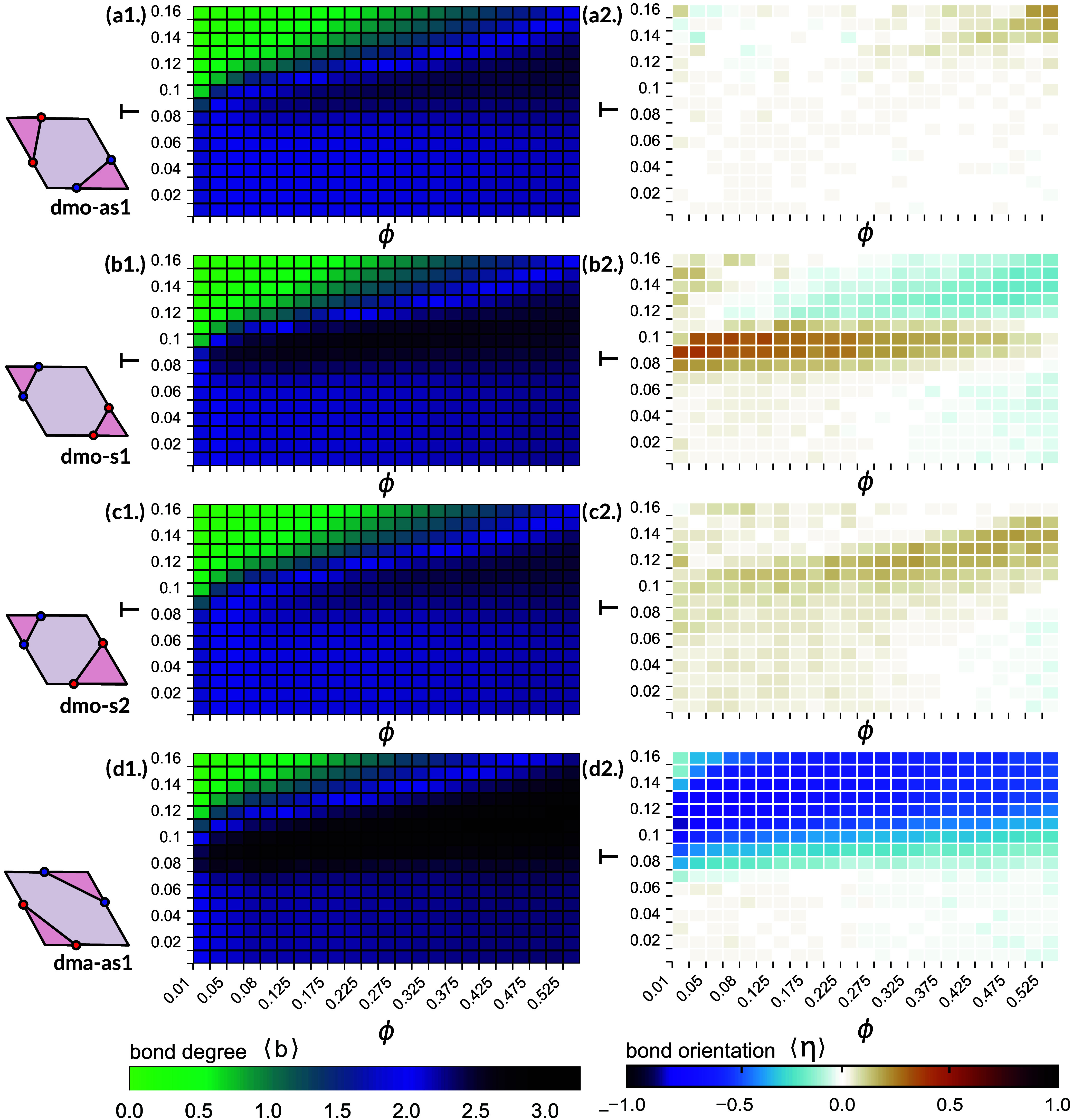
Average number of bonded neighbors (left) and average bond orientation (right). Heat maps for the average number of bonded neighbors, ⟨*b*⟩, and the average bond orientation, ⟨η⟩, across all investigated state points (with ϕ = 0.05–0.525 and *T* = 0.01–0.16) for all studied particle types. At each data point averages are obtained according to the standard statistics procedure (see Section III). (a1–d1) Heat maps for ⟨*b*⟩ for dmo-as1 (a1), dmo-s1 (b1), dmo-s2 (c1) and dma-as1 (d1), where the minimum is zero bonds (bright green) and the maximum is four bonds (dark blue). (a2–d2) Heat maps for the average bond orientation ⟨η⟩ for dmo-as1 (a2), dmo-s1 (b2), dmo-s2 (c2) and dma-as1 (d2), where ⟨η⟩ = −1/+1 denotes the extremes where all bonds are nonparallel (dark blue)/parallel (dark orange) and ⟨η⟩ = 0 indicates state points with mixed bonding on average (white).

A first insight into the assembly scenarios of the selected systems is provided by the average number of bonding neighbors per particle, ⟨*b*⟩, along the chosen grid of state points. The results for all patch topologies are summarized in Figure 2a1–d1 via heat maps in the temperature (*T*) versus packing-fraction (ϕ) plane. We observe that, for the highest *T*, at all but the highest ϕ-values, particles bond to 0–1.5 neighbors on average, which indicates a liquid state where particles are either not bonded or bonded within small clusters. At *T* < 0.09, the average bonding is 2.1–2.2 across all packing fractions, which confirms the network character of the assemblies: here, the majority of particles forms linear chains bonding two patches, that are interconnected with a few branching elements characterized by three bonds. The behavior of ⟨*b*⟩ in the highlighted high and low *T*-regimes fully supports the picture provided by our previous investigations of these four patch topologies, which revealed a liquid state at high *T* and–separated by a percolation line–a disordered particle network at low *T*, which we fully characterized by bonding motifs, porosity and percolation loci.^42^ However, the heat maps of ⟨*b*⟩ across temperature and density show very clearly that, for all four patch topologies, there is a region between the liquid and the network state, where the average bonding exceeds the network bonding of 2.2 and reaches values between 2.3 and 3. While these regions of high bonding do significantly differ in extent in the *T* – ϕ plane as well as in the maximum average bonding across the four patch topologies, we observe that, generally, they do occur at intermediate temperatures and at intermediate to high densities. These domains of higher bonding suggest that the assembled structures in this region may be qualitatively different from the liquid at high and the networks at low temperature. Nonetheless, on the basis of the parameter ⟨*b*⟩ only, a characterization of the assemblies cannot be performed, since this higher bonding might equivalently point to ordered crystals, finite clusters or even to just more tightly bonded disordered networks.

In an attempt to clarify further whether these assemblies are ordered or disordered, we evaluate the orientational order of the aggregates by estimating the average bond orientation of all bonded particles ⟨η⟩: as two patchy rhombi can bond either parallel or nonparallel (see Figure 1e,f for illustration), the bond orientational parameter can be calculated using the percentage of either bonding orientation. In this case, we define ⟨η⟩ = 1–2*P*_np_, where *P*_np_ is the fraction of nonparallel bonds, which means that ⟨η⟩ takes values between −1 and 1, where a value of −1 indicates that the assembly is bonded entirely in a nonparallel fashion, while a value of 1 indicates a completely parallel assembly and a value of 0 indicates a mixed assembly with an equal amount of parallel and nonparallel bonds. In Figure 2a2–d2, where we show the full heat maps for ⟨η⟩, shades of blue/orange represent assemblies with more nonparallel/parallel bonding, while assemblies with an equal mix of parallel and nonparallel bonds are indicated by the white color.

While across all dmo-systems network and fluid states predominantly show mixed bonding, in the region of high bonding dmo-s1 and dmo-s2 exhibit a clear tendency toward either parallel or nonparallel bonding (Figure 2 b2,c2), while dmo-as1 remains of mixed bonding also in the high bonding zone (Figure 2a2). Notably, in the dma-as1 system, a pronounced preference for nonparallel bonding is evident even in the fluid state, persisting through the region of high bonding and changing to mixed bonding only for the disordered network state (Figure 2d2). It is worth noting, that, while the observed bond orientational order for dmo-s1, dmo-s2 and dma-as1 may stem from crystallites with orientational and positional order, it could be also due to small clusters with repeating bonding motifs, either within a fluid state or bonded within an otherwise disordered network. Vice versa, the predominantly mixed bonding for dmo-as1 may indicate an orientationally disordered assembly, but could also signify orientational order with a more complex bonding pattern. We conclude that, while the heat maps for ⟨*b*⟩ and ⟨η⟩ suggest the presence of assembly scenarios distinct from the previously studied liquid and network states, only a broad visual analysis followed by a rigorous crystal structure detection would bring clarity whether these assemblies are ordered or disordered.

### I.II Frustration-Induced Polymorphism

**Figure 3:**
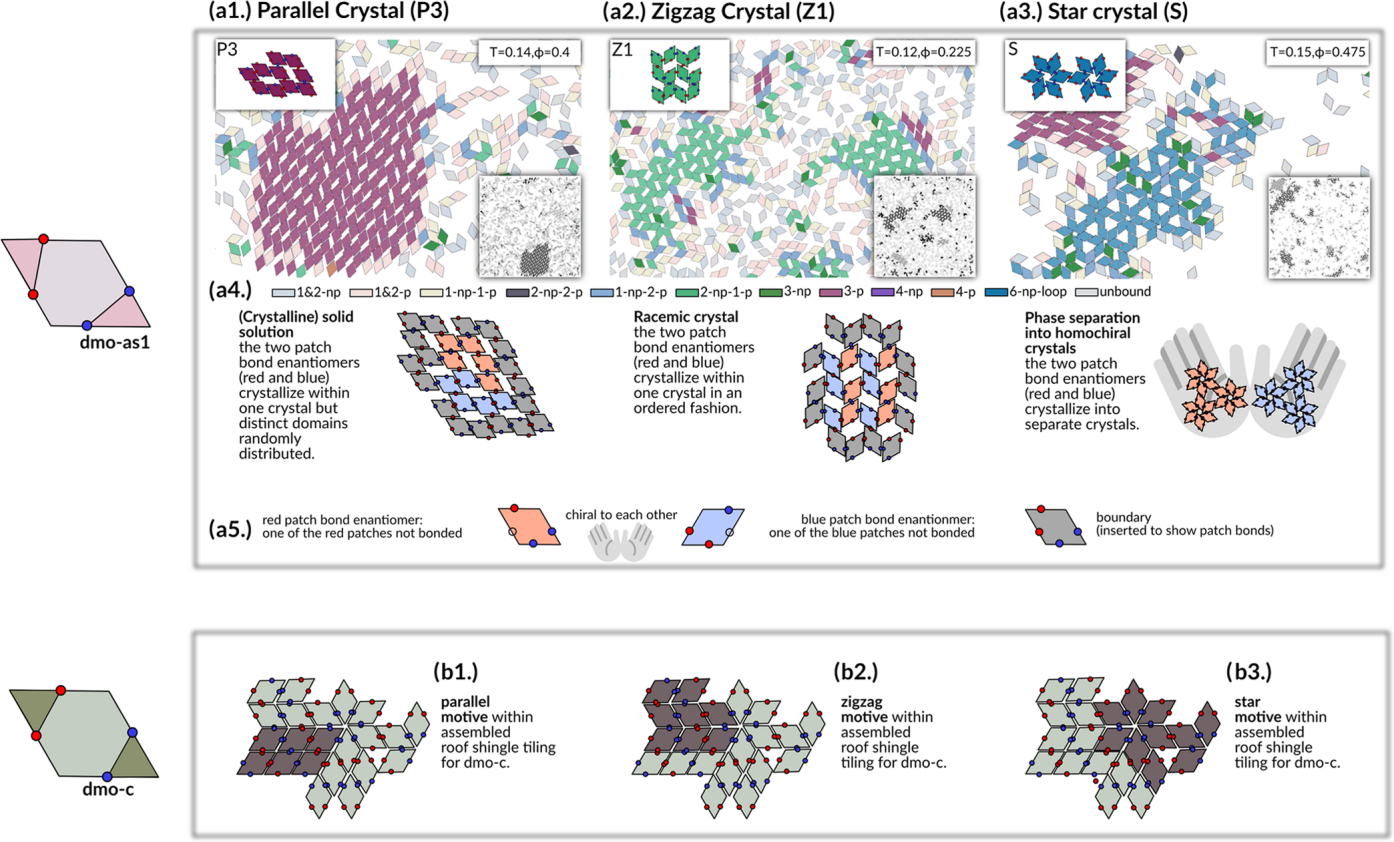
Crystal polymorphs (top) emerging from the geometric frustration of a close-packed tiling (bottom). Snapshots and sketches of emerging crystal polymorphs for dmo-as1 (a-panels) in comparison to bonding motifs in the roof-shingle tiling observed for dmo-c (b-panels), where all patches are in the center of their respective edge.^29,42^ Particles within the simulation snapshots are colored according to the number and orientations (parallel, p, or nonparallel, np) of their bonds. The sketches below the snapshots highlight that all depicted crystals are formed by particles with three out of four patches bonded: either two blue and one red bond (red particles) or two red and one blue bond (blue particles); red and blue particles are chiral to each other (see legend in a5). (a1) Parallel crystal (P3), where each particle has three p-bonds, snapshot taken at ϕ = 0.4, *T* = 0.14. The sketch below the snapshot illustrates that P3 is a solid solution of red and blue particles. (a2) Zigzag crystal (Z1), where each particle has two np- and one p-bond, snapshot taken at ϕ = 0.225, *T* = 0.12. The sketch below the snapshot shows that Z1 is a racemic crystal with an equal amount of red and blue particles. (a3) Star crystal (S), where each particle has two np- and one p-bond, where the np-bonds are arranged in a loop of six np-particles (star), snapshot taken at ϕ = 0.475, *T* = 0.15. The sketch below the snapshot shows that S appears in two homochiral forms—one form containing only the red particles and one only containing the blue particles. (a4) Legend for coloring of particles in the snapshots. The naming generally follows the scheme a-np-b-p, where a is the number of np-bonds and b is the number of p-bonds. Exceptions are 1- and 2-np/p, which refers to particles with only one or two np-/p-bonds, and 6-np-loops, which refers to particles within a closed loop of six np-particles. (b1) Sketch highlighting the fully bonding parallel motif within the observed roof-shingle tiling for dmo-c. (b2) Sketch highlighting the zigzag motif with the observed roof-shingle tiling for dmo-c. (b3) Sketch highlighting the star motif within the observed roof-shingle tiling dmo-c.

**Figure 4:**
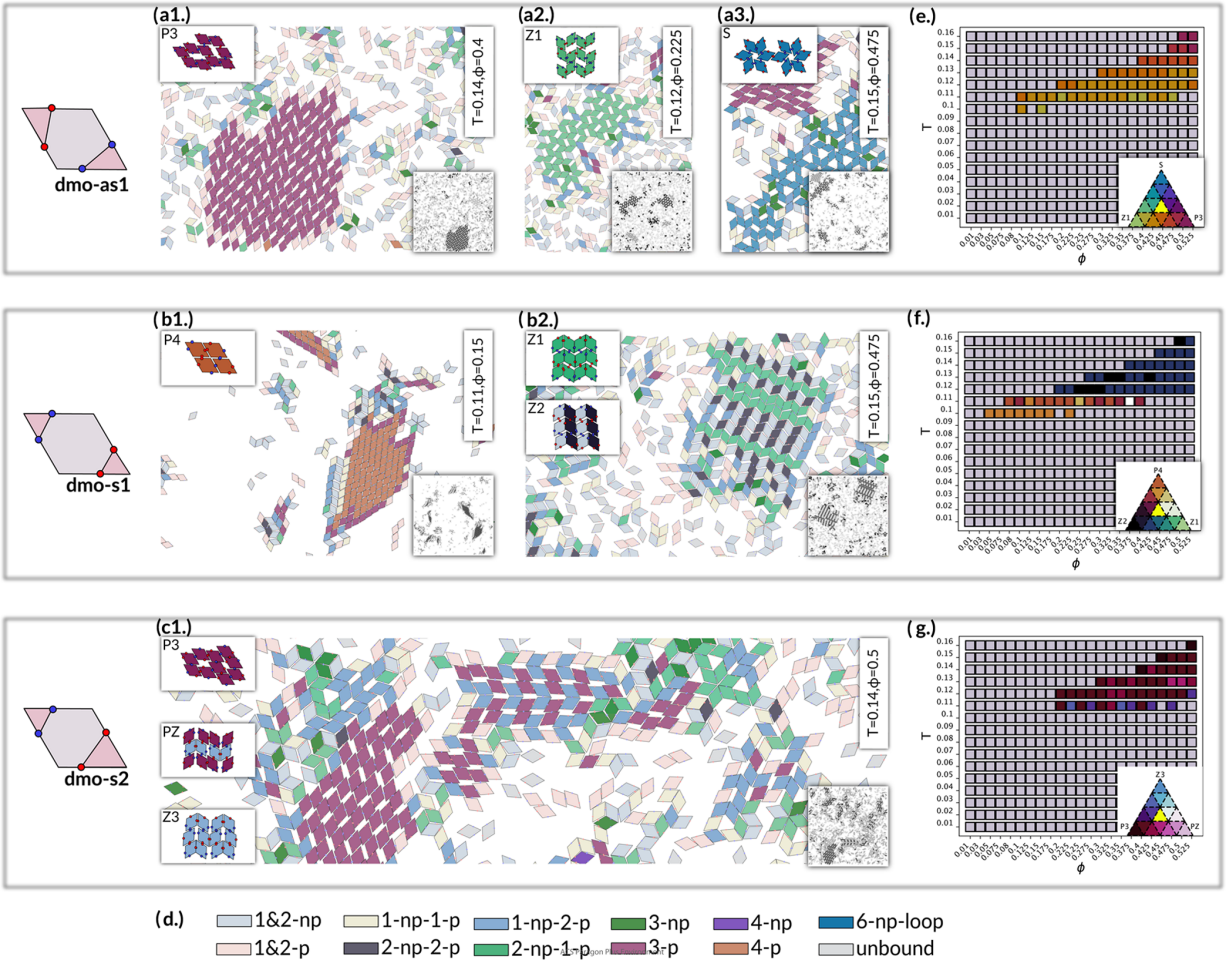
Overview of the observed crystal polymorphs and their relative abundance. (a–c) Snapshots and sketches of emerging crystal structures for dmo-as1, dmo-s1 and dmo-s2 as well as (e–g) heat maps quantifying their relative abundance across different state points (ϕ,*T*). Particles in snapshots are colored according to the number and orientation of bonded neighbors (see legend in (d) with the labeling scheme reported in Figure 3), the corresponding values of ϕ and *T* of each snapshot are reported by the labels. The crystal lattices are sketched at the left corner of each snapshot, while at the bottom right corner the corresponding zoomed-out, gray scale snapshot is reproduced. (a) dmo-as1: (a1) parallel crystal P3, (a2) zigzag crystal Z1, (a3) star crystal S. (b) dmo-s1: (b1) fully bonded parallel lattice P4, (b2) zigzag lattices Z1 and Z2. (c) dmo-s2: (c1) Parallel lattice (P3) and zigzag lattices PZ and Z3. (e–g) Heat maps showing the relative abundance of the three most dominant crystal structures for each particle type across all state points (ϕ = 0.01–0.525, *T* = 0.01–0.16). Relative percentages were obtained by the crystal structure detection algorithm and averaged according to the standard statistics procedure (see Section III). Color mapping is conducted via a the ternary color maps on the lower right corner, where gray indicates state points where percentage of crystalline particles of any kind is below 0.05. For each data point the averages were obtained according to the standard statistics procedure (see Section III). (e) Relative abundance heat map for most dominant crystals (e) P3,Z1 and S for dmo-as1, (f) P4, Z2 and Z1 for dmo-s1, (g) P3, PZ and Z3 for dmo-s2.

We therefore visually inspect the simulation snapshots from all available state points for all four systems and conclude that for three of them–the dmo-systems–we do observe clusters of orientational and positional order–i.e crystallites–in the state point regions of higher bonding. We visually detect three crystal polymorphs per particle type for a total of nine different crystals and we report them all in Figures 3 and 4, where Figure 3 discusses the three polymorphs of dmo-as1 in detail, and Figure 4 shows an overview of all found crystal structures. At this point we like to stress that these reported crystallites are not kinetically arrested; rather, we observe that bonds and even clusters below a certain size form and dissolve in the region in temperature and density where crystallites appear. Additionally, to specifically avoid kinetic traps in the intermediate temperature range, we employed cluster moves throughout the simulations.^2^ Kinetic arrest was only observed in the studied rhombic systems at much lower temperatures (*T* < 0.09). These states were examined in detail in ref (3), where we identified disordered networks.

The majority of these crystals is novel and, to the best of our knowledge, has never been reported before. Therefore, in the absence of an established local order parameter to characterize these novel crystal structures, we craft our own measure. Inspired by the heat maps in Figure 2, where both, ⟨*b*⟩ and ⟨η⟩ show a characteristic variability in the intermediate temperature region of interest, we calculate the number of parallel (p) and nonparallel (np) bonds for each particle and color the snapshots in Figures 3 and 4 accordingly. All occurring bonding patterns and their respective coloring can be found in the legends in the Figures 3a4 and 4d. The resulting colored snapshots show that the chosen order parameter already successfully distinguishes between ordered domains, where the total number of bonds per particle tends to be higher as indicated by a darker color, and disordered domains, where the number of bonds tends to be lower–represented by lighter colors. For this reason, the number of p- and np-bonds per particle will serve as the basis for the crystal structure detection, to which we come later when we quantify the crystallinity of the assemblies and the relative abundance of the polymorphs for different state points.

By analyzing the colored snapshots for dmo-as1 (see the example reported in Figure 3) we identify a parallel lattice P3 (in burgundi), a zigzag lattice Z1 (in green) and a star lattice S (in blue). Despite being characterized by higher bonding, the emerging crystals for the dmo-as1 system are not fully bonded. In fact, due to occurring overlaps when attempting to bond all four patches they cannot form fully bonded assemblies. Instead, all three occurring polymorphs are what we refer to as partially bonded by design, where the crystal lattices are built up from only a subset of all available patch bonds, while the rest of the patches remains unbonded. In the case of dmo-as1 only three out of four patches per particle contribute to the crystal lattices. In Figure 3a1 we observe a large cluster of P3 which is built up from particles connected by three parallel bonds (3-p). The absence of the fourth bonded neighbor creates space and uniform rhombic pores form within the P3 lattice. Similarly, the other two polymorphs of dmo-as1, the Z1 and the S lattices, also consist of three contributing bonds per particle, and again, the lack of the fourth neighbor particle results in the formation of regular pores. Although the bonding pattern of the Z1 and the S lattice is the same with two np-bonds and one p-bond, the lattice structures differ, with the Z1 lattice essentially consisting of np zigzagging rows connected to each other with p-bonds opening up the space for rhombic pores, while the S lattice is built up by 6-particle np-loops–stars–connected to each other by p-bonds, giving rise to large triangular pores between the stars and smaller, hexagonal pores in the center of the stars.

All the crystals emerging in the dmo-as1 system are partially bonded with only three out of four patches per particle forming a bond, where the patch that remains unbonded varies. In the sketches below the snapshots, we highlight this variation in bonding by coloring the particles: in red when two blue patches and one red patch are bonded, in blue when two red patches and one blue patch are bonded. The red and the blue particles are not identical but chiral to each other through their bonding pattern (see the legend of Figure 3a5) and thus in the following we refer to them as patch bond enantiomers.

In the P3 lattice, both bond enantiomers coexist within the lattice and bond seamlessly to each other. Due to bonding constraints, particles of one chirality are bonded to at least one neighbor of the same chirality, and thus form rows of homochiral domains, leading to two distinct pore orientations within the assembly. Within the context of organic chemistry, these connected but distinct domains of the red and blue enantiomers are known as (crystalline) solid solutions.^43,44^ In the Z1 lattice the situation is different, as the zigzagging rows are assembled from alternating red and blue patch bond enantiomers, and the rows are connected to each other alternately by red or blue particles. With an equal amount of red and blue enantiomers co-crystallizing, we classify this assembly as racemic crystal.^45^

On the contrary, S crystals consist solely of one type of patch bond enantiomer, with the lattice’s overall chirality determined by the stars. Typically, stars form six-particle nonparallel loops where particles are bonded through only one type of patch. Consequently, if the stars are constructed from blue patches, the lattice comprises particles with two blue patches bonding within the stars, and one red patch bonding to connect the stars in a parallel fashion. This results in a crystal exclusively composed of the red patch bond enantiomer, where stars formed by red patches—meaning blue patch bond enantiomers—cannot attach. These blue and red S crystals are chiral to each other as well, and because they cannot bond to each other, we characterize them as phase-separated homochiral (enantio-pure) crystals.^46^ In summary, within every polymorph of dmo-as1, partial bonding leads to chiral variations in bonding patterns. Analogously to organic compounds, these chiral bonding patterns give rise to racemic assemblies, that–within a full bonding scenario–could only be realized with binary mixtures of chiral building blocks.^44−46^

Having examined the intricate bonding patterns arising within each polymorph, we now focus on how a single building block–in this case the dmo-as1—can give rise to three different crystalline polymorphs. We find that this problem is best investigated through a comparison with the bonding motifs observed in dmo systems where all patches are placed at the center of each edge (referred to as dmo-c in Figure 3b1–b3). In this case, particles assemble into a close-packed lattice labeled as roof-shingle tiling.^29^ Within such a lattice, we find full bonding analogs of each dmo-as1 polymorph bonding pattern, as illustrated by the sketches: the parallel motif (Figure 3b1), the zigzag motif (Figure 3b2) and the star motif (Figure 3b3). In dmo-c, the central patch position results in on-edge bonding, aligning the particle edges completely. Consequently, all three bonding motifs are commensurable, leading to a disordered tiling with all three motifs present at once. It is only by slightly shifting the patches off-center to the dmo-as1 patch topology that lattice selectivity arises where the different bonding motifs are not compatible anymore, leading to the formation of distinct polymorphs.

### I.III Relative Abundance of Polymorphs

Up to this point we have characterized the emerging assemblies solely by the number of parallel and nonparallel bonds of the single particles. As this measure separates well between particles within crystalline aggregates and particles bonded in disordered domains, we use it as the basis for crystal structure detection. To do so, we initially determine the bonding pattern characteristic of the crystal structure under consideration. Subsequently, we classify a particle as part of this particular crystalline environment if the particle itself and at least one neighbor display the specific bonding pattern associated with the crystal. As an example, we consider the P3 lattice of dmo-as1, where the local P3-bonding pattern consists of three parallel bonds. In this case, a particle is considered P3-crystalline if the particle itself and at least one bonded neighbor have three parallel bonds. For all other crystals, the structure identification is in principle analogous, and we refer to the Supporting Information for details.

To determine the system-wide prevalence of a particular crystal structure, we compute the ratio of the number of particles detected to be of this crystal type to the total number of bonded particles. This yields a value ranging from 0, indicating the complete absence of this specific crystal structure in the assembly, to 1, signifying that all bonded particles in the simulation box are part of this specific crystal type. This metric allows us to compare the prevalence of different polymorphs within a single assembly and, when averaged for each state point, across all state points. We summarize the results of the crystal structure analysis for all dmo-systems in the heat maps presented in Figure 4e–g, along with simulation snapshots showcasing the three most prevalent crystal structures. In the heat maps the relative prevalence of the polymorphs is represented by a single color, using a barycentric color space (depicted in the lower right corner of the heat map). As each dmo system has three dominant polymorphs, we define a barycentric triangle whose edge points are associated with the three polymorphs, where, for ease of reading, the corner colors match the colors of the respective crystals in the snapshots.

We note that this analysis is fully automated, from the identification of the crystalline environments to the plotting of the heat map with barycentric coloring method. If a state point is colored in one of the corner colors of the barycentric triangle it indicates that one of the three crystals dominates over the other two. Conversely, if a state point exhibits a more mixed assembly, colors in between are used.

We start our discussion with the dominant crystals of the dmo-as1 system (see Figure 4a1–a3). The quantitative results of this analysis are presented in the heat map in Figure 4e. In this heat map only state points with an overall crystallinity above 0.05 are shown in color, while state points below this threshold are grayed out. This analysis reveals that dmo-as1 displays significant crystallinity for packing fractions above 0.1, where the extent in temperature of the crystalline region increases with higher packing, from including only *T* = 0.1–0.11 for ϕ = 0.1 to including the whole upper range of temperatures from *T* = 0.10–0.16 for the highest packing fraction of 0.525. With the barycentric color map at hand, we clearly observe that while for lower temperatures and packing fractions, mostly Z1 and P3 crystallites are competing (as indicated by yellow/orange), the P3 lattice becomes more prevalent and eventually emerges as the dominant crystal structure at the highest temperatures and packing fractions (indicated by burgundi).

By analyzing the colored snapshots for dmo-s1 (see the examples reported in Figure 4b1–b2) we identify a fully bonded parallel lattice (P4) and two partially bonded zigzag lattices (Z1 and Z2). It is worth stressing that, in contrast to all other three particle types, for dmo-s1 full bonding is possible without overlaps or energetic penalties. Nonetheless, bonding of all four patches results in a certain degree of strain, as the patches are almost out of each other’s interaction range and barely reach to bond (see Figure 1h for an illustration of bond strain). The other two prevalent lattices are partially bonded: while at first sight they seem equivalent, Z1 has a 2-np-1p bonding pattern–bonding to three neighbors–while Z2 displays two alternating bonding patterns, where in one pattern the particles bond to two neighbors in a nonparallel fashion (2-np), while in the other pattern the particles bond to four neighbors with two nonparallel and two parallel bonds (2-np-2p). On the level of lattice geometry, these bonding pattern differences show in that, while Z1 connects the zigzagging rows with vertically alternating p-bonds, Z2 connects them with vertically aligned p-bonds. From the aspect of chirality/number of effective components, the Z1 of dmo-s1 is a racemic co-crystal with the same enantiomer arrangement as the Z1 of dmo-as1, while Z2 is an effective symmetric binary lattice of two- and four-patch particles. That said, it must be noted that Z1 and Z2 are separated only by a horizontal shift in bonding between the zigzag rows. Additionally, Z1 and Z2 are compatible with each other and often co-occur as can be also observed in the snapshots in Figure 4b2.

Analogously to dmo-as1, also for dmo-s1 we conduct the crystal cluster analysis resulting in the automatic plotting of the heat map that quantifies the relative abundance of the dominant crystals (see Figure 4f). Inspecting the region of overall crystallinity in the heat map, we find the crystalline region to be at intermediate to high temperatures/packing fractions, similar to dmo-as1, with the distinction that it extends a little further to lower densities (up to ϕ = 0.05 *versus* ϕ = 0.1 for dmo-as1). When comparing the prevalence of the three dominant lattices, we find that, at low *T* and ϕ, P4 clearly dominates over Z1 and Z2, while, as we move to higher *T* and/or ϕ there is an intermediate region where P4 competes with Z2 and–to a lesser degree–with Z1. Finally, for higher *T* and ϕ, Z2 dominates with some degree of Z1 present for some state points. The described heat map shows the counterintuitive observation that the close-packed and fully bonded P4 crystal is prevalent at lower packing, while the porous and partially bonded Z2 lattice prevails at high-packing. We rationalize this observation by noting that, for lower packing fractions, the crystalline region resides also at lower temperatures, where the drive to fully bond all available patches is larger (despite the unavoidable strain). For higher packing fractions the crystal region is found at higher temperatures, meaning that the drive for strained full-bonding is reduced and thus the Z2 lattice prevails.

**Figure 5:**
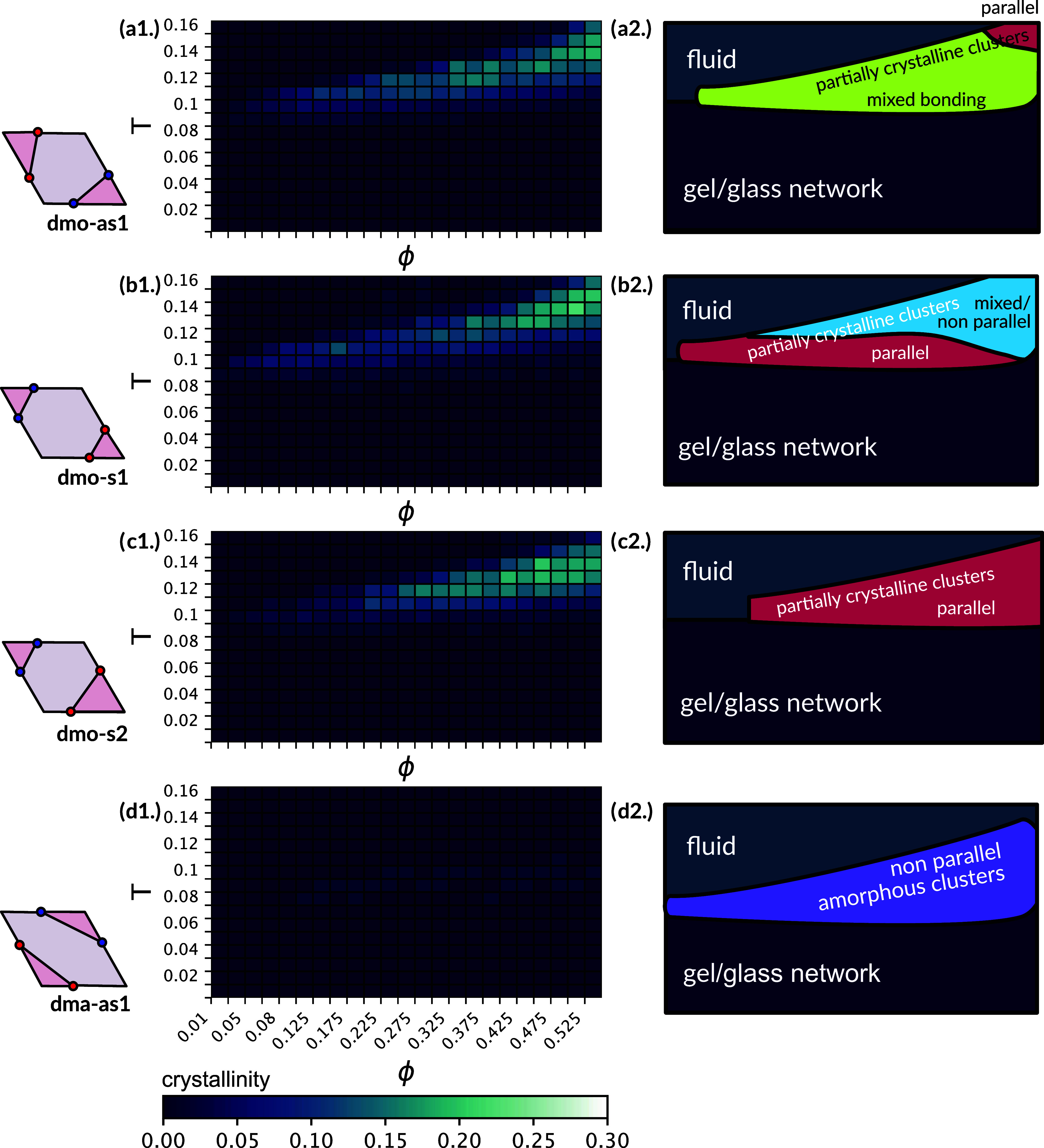
Absolute abundance of crystal polymorphs (left) and overview of the investigated state diagrams (right). Heat maps (a1–d1) for the overall average crystallinity, ⟨ξ⟩, for all studied particle types across all state points (with ϕ = 0.01–0.525 *T* = 0.01–0.16), and sketches (a2–d2) for the dynamic state diagrams. Crystallinity heat map for (a1) dmo-as1, (b1) dmo-s1, (c1) dmo-s2, (d1) dma-as1. Dynamic state diagram for (a2) dmo-as1, (b2) dmo-s1, (c2) dmo-s2, (d2) dma-as1.

**Figure 6:**
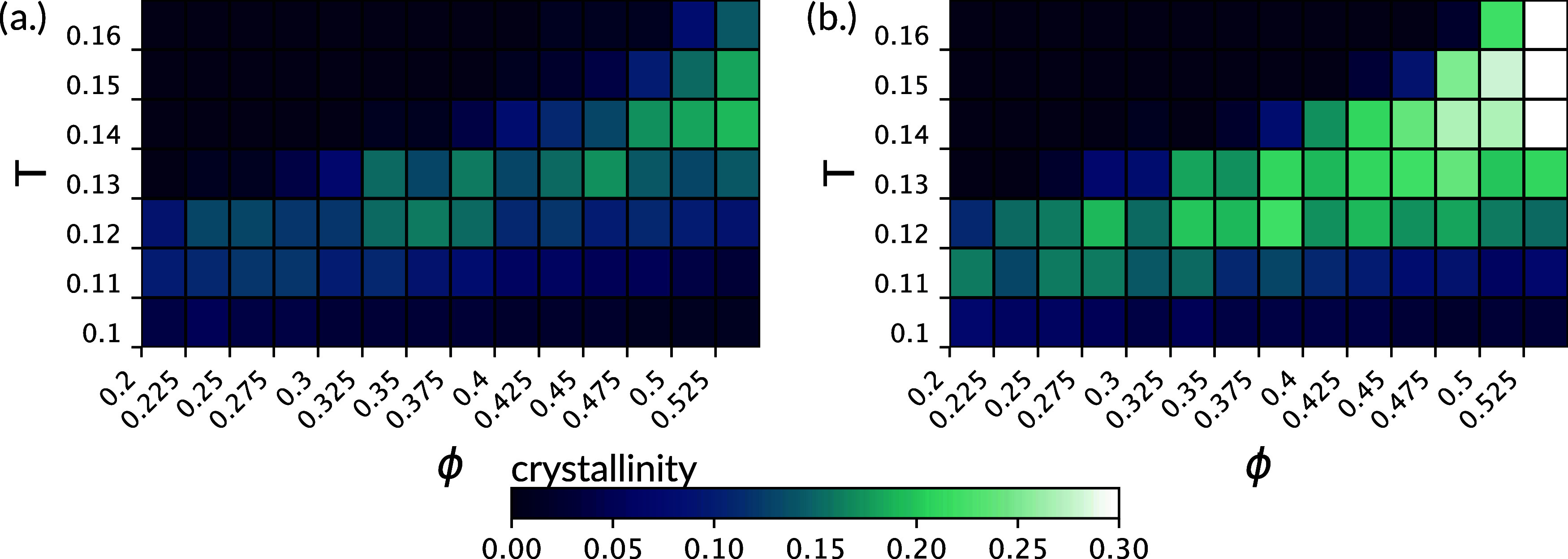
Comparison of overall crystallinity for dmo-as1 systems at different packing fractions ϕ and temperatures *T* for (a) shorter runs (a zoom in of the heat map depicted in Figure 5a1) at MC sweeps ≈2.5 × 10^7^ and (b) longer runs at MC sweeps ≈3.5 × 10^7^.

In dmo-s2, all emerging crystals exhibit partial bonding with three bonds per particle (see Figure 4c1). The P3 lattice (particles in burgundi) is nearly identical to the P3 lattice found in dmo-as1, with the fine distinction that the dangling patch is always a red one. As it varies which of the two red patches is dangling, the bonding pattern still yields two patch bond enantiomers, leading to an equivalent solid solution, with again two distinct pore orientations in the assembly. The PZ lattice features a bonding pattern that alternates between particles with 3-p bonds (in burgundi) and particles with 2-p and 1-np bonds (in blue), making it an effective binary mixture of two particles with different patch arrangement. In both particle bonding patterns, it is always a red patch that is left dangling. The PZ geometry can be viewed as a lattice where parallel rows of p-bonded (alternately burgundi and blue) particles are alternately connected to each other with p- and np-bonds. The third dominant lattice for dmo-s2 is Z3, where all particles 2-p and 1-np bonds. Z3 lattice connects parallel rows of p-bonded particles with np-bonds only. Looking at the dominance across all state points, we find that, while P3 dominates most of the state points, for some state points we observe a substantial amount of PZ and Z3 environments–highlighted by pink (more PZ) and blue (more Z3) shades. In the dmo-s2, the crystalline region follows the same trends as the other dmo-systems, with the crystalline region extending from only including a small intermediate *T*-range at low ϕ to encompassing the whole upper *T*-range for the highest ϕ. We note that, with respect to the previous dmo systems, the crystalline region is reduced in density, while its stays very similar in temperature: it starts at ϕ = 0.2 (*versus* ϕ = 0.1 for dmo-as1 and ϕ = 0.05 for dmo-s1) and at *T* = 0.11 (*versus T* = 0.1 for both dmo-as1 and dmo-s2). As already observed with dmo-as1, for the highest packing fraction, the system clearly exhibits the P3 lattice. For dma-as1, two crystalline domains are observed (see the SI for details) but, as the overall crystallinity remains below 5%, we exclude these systems from further analysis. The visual inspection of the colored snapshots reveals that the high np-bonding observed in Figure 2 is not due to an emergent crystalline order but rather to the abundance of finite-sized, three particle clusters, embedded in a disordered network (see the SI for details). Finally, we evaluate the absolute amount of crystallinity, ⟨ξ⟩, as the ratio between the number of particles within any crystalline structure (according to the structure detection algorithm) and the number of bonded particles, averaged according to the standard statistics procedure (see Section III). Interestingly, the overall crystallinity remains low over an extensive simulation time: for most systems, ⟨ξ⟩ is highest at 20%, as reported in Figure 5(a1–d1). Note that this a conservative estimate, as we do not include the domain boundaries of the crystallites. Nonetheless, the low degree of crystallinity raises the question of which mechanism hinders the crystal growth, whether it is a matter of stability or instead due to the competition between the frustrated polymorphs.^47−50^ To gain insight into this issue, we extend our simulation runs for the dmo-as1 system from 2.5 × 10^7^ sweeps to 3.5 × 10^7^ sweeps and compare the resulting overall crystallinity in Figure 6. While the region of the state diagram where crystallites are observed remains the same for short (left) and long (right) simulations, the overall crystallinity grows beyond 30% (50% in some cases) for larger packing fractions, but stays the same for lower packing. It is worth stressing that the overall crystallinity is defined as the percentage of crystalline structures within the bonded assemblies, meaning that the aggregates are partially disordered, both at high and low packing fractions. An inspection of the snapshots at different packing fractions (see Figures 7 and 8 of the SI) confirms that no particle depletion hinders the growth of the crystallites, at high as well as low packing fractions. Finally, we perform large-scale simulations for selected state points of the state diagram of dmo-as1 to check whether the size of the crystallites is limited by the system size: our results show that the overall crystallinity is not affected by the size of the system (see Figure 9 of the SI). The observed scenario suggests that, while the partially bonded crystallites are not fundamentally limited by frustration, they take an excessively long time to grow, possibly because of the many bonding possibilities, including competing polymorphs. To better understand this, more investigations such as free energy calculations and free energy barriers are needed.

## II Conclusions

While design and inverse design strategies rely on optimizing particle shape, functionalization and bonding geometry in order to access complex mesoscopic phases, we purposefully introduce geometric frustration in systems of building blocks designed to otherwise assemble in well-defined monolayers. In contrast to the guiding principle of the most recent approaches to target specific structures, which is to satisfy all possible bonds between the assembling units, we deliberately disfavor such a condition by introducing a finite set of possible full bonding failures: particle overlaps, bonding strain, bonding incompatibility and energetic penalties. When partial bonding is favored, disordered aggregates often emerge as a result of geometric frustration. Nonetheless, our investigation clearly shows a different route to crystallization where geometric frustration can be utilized to target complex structures. We summarize our results in a dynamic state diagram, reported in Figure 5(a2–d2). In the state diagram of all investigated systems, we observe a pocket of state points–between the low-temperature disordered network region and the high-temperature/low-density fluid–where particles have a high-bonding degree. In this region, we observe the emergence of crystallites with different, but energetically equivalent, bonding patterns for all dmo systems. We stress that the found polymorphs are the ones that are accessible from the fluid state. In order to determine the relative stability of these polymorphs and with respect to the fluid state free energy calculations would be necessary. The assembly scenarios of those systems have some non-negligible differences with respect to each other.

For dmo-as1, we observe both parallel and nonparallel bonding in the whole high-bonding region, emerging from the tight competition between two (out of three) porous polymorphs, where the parallel lattice eventually prevails over the zigzag one only at high temperatures and densities. In contrast, for dmo-s1, we observe that the region of high bonding has a prevalence of parallel bonding at low temperatures and densities, related to the prevalence of the only close-packed, fully bonded (but strained) parallel lattice, and a region of mixed parallel and nonparallel bonding at higher temperatures and densities, due to the prevalence of two zigzag competing crystals. Finally, for dmo-s2, we observe a prevalence of parallel bonding, emerging from the competition between three porous polymorphs, across the whole domain of crystallinity. In contrast to those systems, for dma-as1, the amount of crystallinity in the region of high-bonding degree is very low and thus the strong nonparallel pattern features of the assemblies must be attributed to the formation of a large number of finite three-particle clusters, connected within a disordered network.

Our results show that a different paradigm can be leveraged to build up ordered porous structures from patchy colloids, where the full bondedness of the particles is not anymore a requirement for crystallinity. The same paradigm can be used for molecular tilings. While extended monolayers of small organic molecules have been reproduced by fully bonded, two-dimensional lattices of patchy platelets,^28,29,51^ we show that the presence of dangling bonds can produce extended crystals, suggesting that geometric frustration could be used as a guiding principle to gain a deeper understanding of organic chemistry mechanisms. Our thorough analysis of the crystal polymorphs in the presence of geometric frustration highlights the emergence of chiral building units, leading to solid solutions, racemic crystals and homochiral crystals. In fact, the proposed paradigm based on polymorphism, chirality and dangling bonds play a key role in the design of bioactive organic compounds, for instance in industrial applications, where homochiral (or enantiopure) crystals are in high demand.^50,52−57^ Finally, we believe that we are only beginning to understand the effects of frustration in colloidal crystals. Future work should take into account the effect of diverse polygonal shapes and further explore the link between frustration and the interplay of shape and patchiness.

## III Methods

### III.I Particle Model

For this self-assembly study we consider regular hard rhombi with four localized patches–one per edge–of two distinct types, where pairs of patches are of the same type and where same-type patches attract and different-type patches repel each other. This type of building block assembles into a plethora of distinct porous and close-packed assemblies upon varying the exact placement of the four patches on the rhombus edges.^29,41,42^ In this work we focus on a subset of systems, where the bonding is geometrically frustrated: we deliberately select four patch layouts where all four patches cannot be simultaneously bonded to neighboring particles without incurring overlaps, energetic penalties or at least a certain degree of strain. Bond strain occurs whenever bonded patches can not fully overlap and therefore bonded particles cannot explore a large set of bonded orientations/positions. All four particle types are shown in Figure 1(a–d), together with example motifs where the full bonding fails in Figure 1(g–j). The particle naming scheme leans on previous nomenclature from ref (29), where dmo refers to systems where the patches of the same type enclose the vertex of the small rhombus angle (Figure 1a–c) and dma (Figure 1d) refers to systems where the patches of the same type enclose the vertex of the large rhombus angle.

Furthermore, the suffixes (as) and (s) denote whether same type patches are arranged asymmetrically (as) or symmetrically (s) with respect to the enclosed reference vertex, while the numbers 1 and 2 just serve as identifiers for the different arrangements. We call the combination of these three identifiers a patch topology, hence the four patch topologies of this study are dmo-as1, dmo-s1, dmo-s2 and dma-as1. The last parameter required to uniquely characterize each patch arrangement is the distance of the patches from the edge center Δ*_c_*, which takes values between 0 (patches are in the center) and 0.5 (patches are at the vertices). In other words, Δ*_c_* can be viewed as a measure of patch anisotropy, that indicates how much off-center the patches are placed–or in terms of assembly scenarios–how far away the systems are from close-packing at Δ_*c*_ = 0.^29^ While full bonding is disfavored for all off-center Δ_*c*_ with 0 < Δ_*c*_ < 0.5 for all four patch topologies,^42^ we select Δ_*c*_ = 0.1 for this investigation to highlight the impact a slight patch anisotropy on the self-assembly.

The interaction between two rhombic platelets *i* and *j* is characterized by a hard-core potential
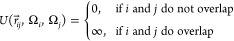
where *r⃗*_*ij*_ represents the center-to-center distance vector, and Ω_*i*_ and Ω_*j*_ denote particle orientations. Overlaps between two rhombi are determined via the separating axis theorem.^58^ The side length of the rhombi, *l*_*r*_, is set to 1. Patch-to-patch interactions are defined by an attractive or repulsive square-well potential
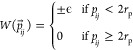
where *p*_*ij*_ represents the patch-to-patch distance, 2*r*_p_ is the patch diameter, and ϵ denotes the patch interaction strength. We set *r*_p_ = 0.05 to ensure that each patch can bond at maximum to another patch^59^ and ϵ = ±1.

### III.II Simulation Details

We investigate the self-assembly of these rhombic building blocks using large scale real-space Monte Carlo simulations in two dimensions incorporating single particle rotation and translation moves, alongside cluster moves.^60,61^ With a fixed particle count of *N* = 1500, a grid of state points is constructed: *m* = 16 temperatures (*T*_0_ = 0.01 to *T*_*m*_ = 0.16) and *n* = 24 packing fractions (ϕ_0_ = 0.01 to ϕ_*n*_ = 0.525), totaling 384 state points per system. The initial state for all systems is a square lattice of rhombi, that is melted/equilibrated for 1 × 10^6^ Monte Carlo sweeps by switching off the patch interactions, which is equivalent to simulating at infinite temperature. The self-assembly process is then started by switching on the patch interactions and setting the temperature according to the value of the respective state point. For consistency, we use the same simulation protocol for all systems across various states, performing a substantial total of 24,576 separate simulations considering all 16 parallel runs and 384 state points across all 4 particle topologies. Expecting the slow formation of disordered particle networks we conduct long simulations extending to 2–2.5 × 10^7^ Monte Carlo sweeps. The statistics for each state point in temperature/packing fraction is aggregated by including the last 100 checkpoints of all 16 parallel runs, where checkpoints are recorded every 10,000 sweeps. Within this work, we refer to this choice of averaging as standard statistics procedure.

## Supporting Information Available

Details on the crystal structure detection from bonding patterns for all systems as well as crystallinity calculations and snapshots for longer and larger runs for the dmo-as1 system (PDF)
