# Supplementary material for: Partially Bonded Crystals: A Pathway to Porosity and Polymorphism
Source: ACS Nano. 2025 Jan 28;19(5):5146–57. doi: 10.1021/acsnano.4c06489 (PMC11823632; doi:10.1021/acsnano.4c06489)
Supplement: Supplementary file 1 — nn4c06489_si_001.pdf [file nn4c06489_si_001.pdf]

Supporting Information – Partially bonded crystals: a pathway to porosity and polymorphism

Carina Karner*

Institut für Theoretische Physik, TU Wien,

Wiedner Hauptstraße 8-10, A-1040 Wien, Austria

Emanuela Bianchi†

Institut für Theoretische Physik, TU Wien,

Wiedner Hauptstraße 8-10, A-1040 Wien,

Austria and CNR-ISC, Uos Sapienza,

Piazzale A. Moro 2, 00185 Roma, Italy

I. CRYSTAL STRUCTURE IDENTIFICATION

The four patch topologies featured in our work – dma-as1, dmo-as1, dmo-s1 and dmo-s2 – all give rise to a set of crystal polymorphs with distinct symmetries.

Bonding pattern identification. We find that these polymorphs can be most effectively distinguished from each other via their bonding patterns. In this case we define a bonding pattern to be the number of parallel (p) and non-parallel(np) bonded neighbours of the particle. After the visual inspection of all available snapshots, we find in total ten bonding patterns that contribute to the formation of crystalline units. In Figure 1 we show sample snapshots for all four systems, where the particles are algorithmically colored according to these neighbourhoods, where we list all considered bonding patterns in the legend. It is important to note that, for up to three bonded neighbours, the listed bonding patterns encompass all bonding possibilities. In contrast, for four bonds only four parallel bonds (4-p), four non-parallel bonds (4-np) and two parallel plus two non-parallel bonds (2-np-2p) are observed, while bonding patterns with three parallel (non-parallel) bonds and one non-parallel (parallel) bond are never observed as part of a crystallite. Due to the coloring we can already observe, in all systems, some of the crystallites with their typical bonding patterns and symmetries visually distinguished from the liquid around, as the number of bonds is on average three while the average bonding for the liquid is between one and three. By inspecting the snapshots, however, we can also see that not all bonded neighbours have a crystal symmetry. Therefore the next step must be a crystal structure identification that robustly identifies whether a particle is bonded within a crystal neighbourhood and not bonded by chance – at the single particle level – according to a specific bonding pattern.

* carina.karner@tuwien.ac.at
† emanuela.bianchi@tuwien.ac.at

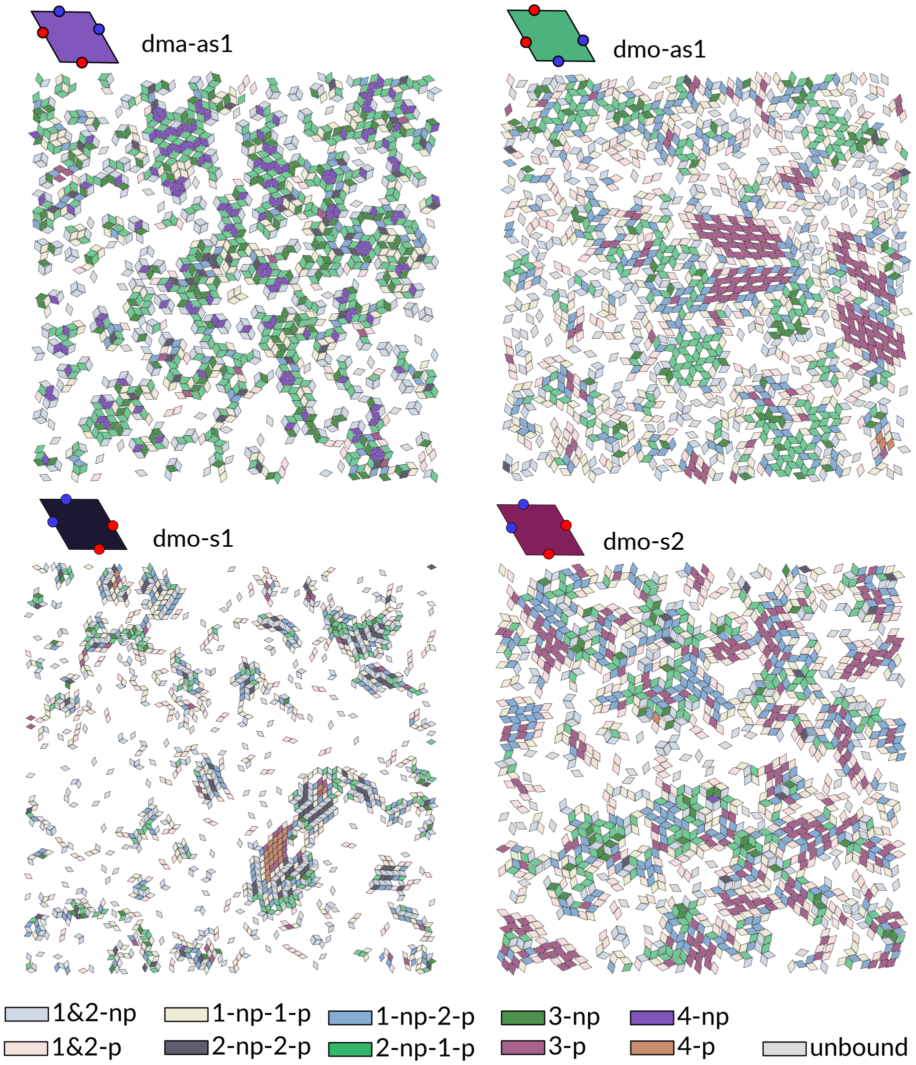

FIG. 1. **Bonded neighbourhoods in sample snapshots of the four investigated systems.** As labelled: dma-as1 ($\phi = 0.45$, $T = 0.13$), dmo-as1 ($\phi = 0.5$, $T = 0.14$), dmo-s1 ($\phi = 0.225$, $T = 0.12$) and dmo-s2 ($\phi = 0.5$, $T = 0.13$). Generally label abbreviations follow the scheme a-np-b-p, where a is the number of non-parallel bonds and b the number of parallel bonds. For example, 2-np-2-p denotes a particle with two non-parallel and two parallel bonds. Exceptions to this scheme are 1&2-np and 1&2-p, that indicate particles with either one or two non-parallel (np) or one or two parallel (p) bonds.

From bonding patterns to crystal structures. In general, for a particle to be seen as part of a crystalline neighbourhood, the particles neighbours have to be arranged in a certain symmetry around the particle, and, additionally the neighbours themselves have to be subject to a neighbourhood of the same crystalline symmetry. Ordinarily, one would therefore rely on symmetry detecting positional order parameters such as the Steinhardt bond order parameters, that take into account distance vectors of the nearest neighbours for crystal structure detection [1]. In this work, however, we refrain from using the Steinhardt order parameters, because they rely purely on center-to-center positions, while our crystals exhibit a complex orientational ordering. Global structure identification methods, such as radial distribution function or structure factor, would prove insufficient because of the presence of competing polymorphs. This means that we would not be able to assign emerging crystal peaks to a specific crystal structure. As we realized that in all but one system – the dmo-as1 system we will discuss later — the set of bonding patterns are unique to each polymorph, we built our crystal detection on top of the highlighted bonding patterns. Specifically, from visual inspection we identified by hand 11 different sets of bonding patterns (in total) as candidates for the crystal structure identification. Here it must be added that, while in principle, it is possible to overlook crystal structures via visual inspection, the highlighting due to the coloring of the bonding patterns and the fact that we thoroughly inspected the outcomes of all simulation runs, re-assures us that we did not overlook pre-dominantly occurring crystal structures. To illustrate how the structure identification process is set up we go through the crystal candidate algorithm for dma-as1. The insets of Figure 2 show sketches of the two identified crystal candidates. The first is the B34 crystal, where zigzagging rows of 4-np particles (in lilac) are connected via particles with 2-np bonds and 1-p bond (in pink), and the second is the NP4 crystal, where the particles are fully bonded with np-bonds (4-np), creating a close-packed, non-parallel monolayer. To classify a particle as crystalline, we essentially follow a pattern matching algorithm, where we define the bonding pattern of the perfect target crystal as well as a threshold to account for crystal defects. Target pattern and threshold are given for B34 and NP4 in the inset of Figure 2, next to the crystal sketches. In the perfect target B34, crystal particles can be in one of two environments. In the first one (case a. in the inset of Figure 2), the particle itself is 4-np bonded, and two neighbours must be 4-np bonded themselves and two neighbours must be 2-np-1p bonded in order for the particle to be classified as B34. In the other case

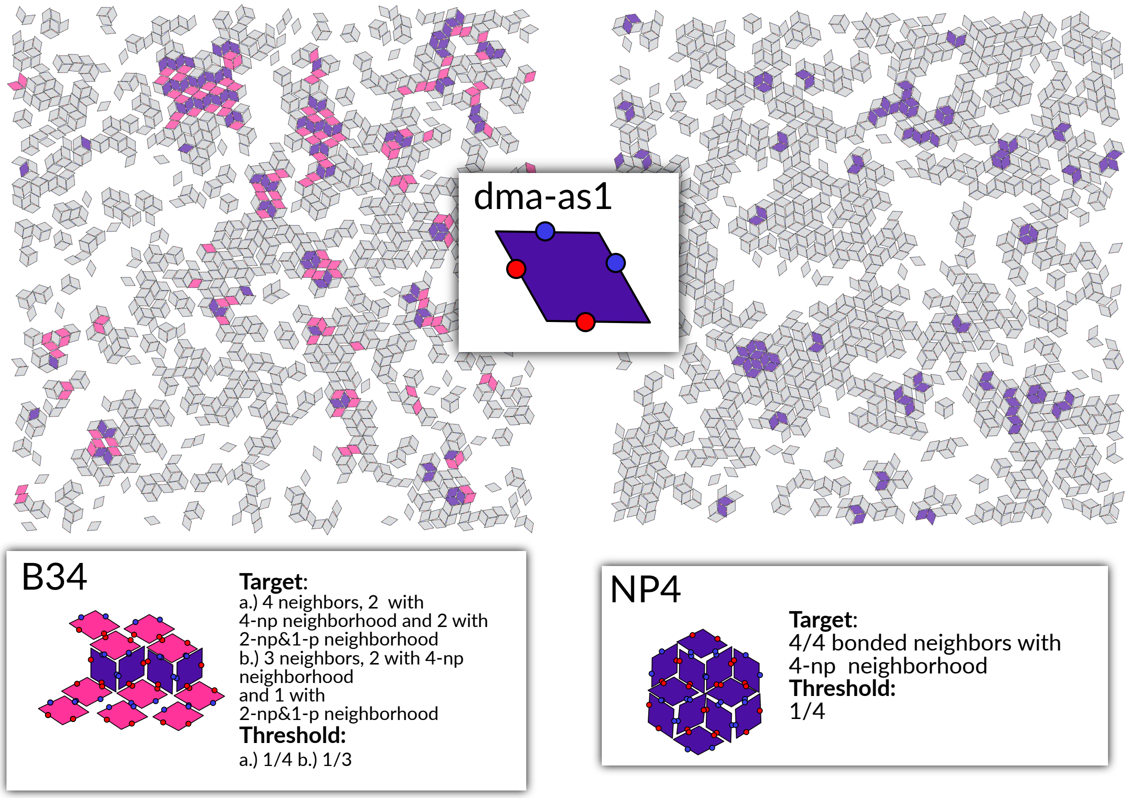

FIG. 2. **Crystal structure detection for dma-as1.** On the left: simulation snapshots at $\phi = 0.45$, $T = 0.13$ with particles detected as B34 particles colored: particles with 4-np bonding pattern in lilac, particles with 2-np-1p in pink. Inset: Target and threshold for B34 structure detection. On the right: simulation snapshots at $\phi = 0.5$, $T = 0.13$ with only particles detected as NP4 crystal colord in lilac. Inset: Target and threshold for NP4 structure detection.

(case b. in the inset of Figure 2), the particle is itself 2-np-1-p bonded, then two neighbours must 4-np bonded and one neighbour must be 2-np-1-p bonded. To allow for a degree of imperfections we set threshold values. To be classified as B34-crystalline a particle must be either (a) 4-np bonded or (b) 2-np-1p bonded and in both cases at least one of the bonded neighbours must be either 2-np-1p bonded or 4-np bonded. In the case (a) that is one out of four bonded neighbours – denoted as 1/4 in the legend of Figure 2 — or one out of three bonded neighbours (1/3) in case (b). The target and threshold instructions for the NP4 crystal can be read equivalently to the B34 structure. For NP4, there is only one target pattern: a particle is defined as NP4-crystalline if the particle itself and all four bonded neighbours are bonded 4-np. Analogously, the threshold requirement is that at least the

particle itself and one neighbour is 4-np bonded (1/4).

To show how well this crystal detection performs, we added two simulation snapshots in Figure 2, where we use the reported target and threshold to automatically identify B34 environments (on the left) and NP4 environments (on the right). We find that, while the larger crystallites are identified correctly, for smaller clusters we observe some mismatches. We solve this problem (for dma-as1 and in general for all other systems) by labeling a particle as crystalline only if it is within a crystal environment larger than 5 crystalline particles. Note that a particle can be a member of two crystal structures, as illustrated in Figure 2, where with the chosen threshold, the 4-np row (in lilac) within the B34 which is both part of B34 and also detected as NP4. We regard this as a feature because it enables us to identify the crystal structure of single defect lines, but it can be in principle alleviated with a stricter threshold. Note, however, that to estimate the total crystallinity ξ – i.e. the total number of all crystalline particles over all bonded particles – we only count whether a particle is crystalline or not and do not double count in cases where a particle is classified as part of two crystal structures.

Overall, the snapshots in Figure 2 illustrate what the automatic crystal structure detection across all simulations runs found: despite the presence of some small B34 and NP4 crystallites larger than 5 particles, the overall crystallinity is low. In fact our calculation over all simulation runs show that the average crystallinity $\langle\xi\rangle$ of dma-as1 is below the threshold of 5% for all temperature and density state points (see Figure 5 in the main text) and we conclude that the self assembly products of dma-as1 are not crystalline in character.

For the other three systems - dmo-as1, dmo-s1 and dmo-s2 – the target and threshold instructions can be read in the same way as for B34 and NP4.

For the dmo-as1 systems, shown in Figure 3, where we find larger crystallites present, particularly for the P3 and the Z1 crystals, the performance of the crystal detection algorithm can be judged more thoroughly. At first, the threshold requirement, that only the particle itself and one neighbour has to adhere to the target crystal structure, may seem weak. However, by visual inspection of the P3 and Z1 systems, we find that the structure detection is in fact rather accurate, an in contrast, turns out to be even quite strict, as boundary particles are not classified as crystalline in most cases, as the particles themselves do not fulfill the bonding pattern - either because they miss bonds or because one or more bonds is mismatched with respect to the required bonding pattern. For the crystal structure detection

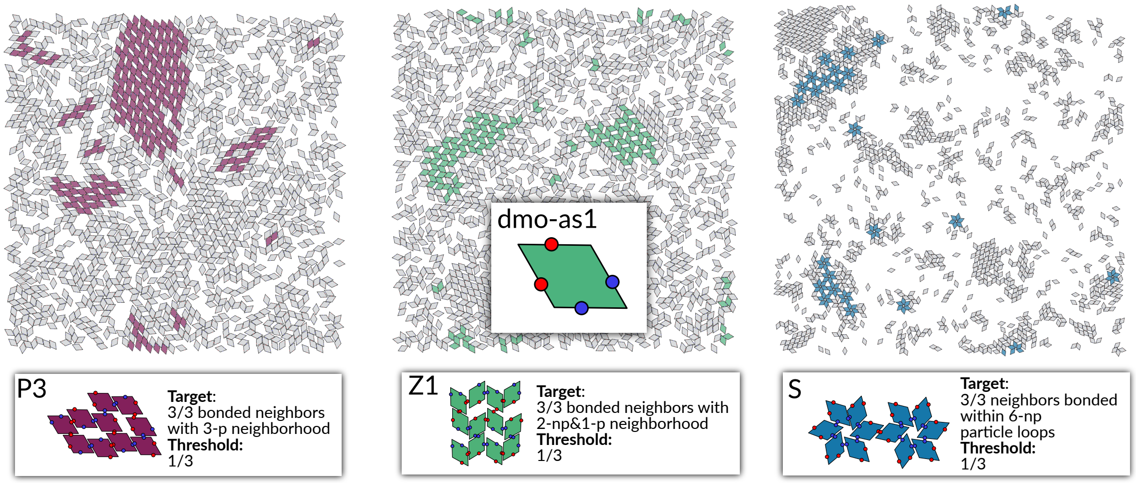

FIG. 3. **Crystal structure detection for dmo-as1.** On the left: simulation snapshots at $\phi = 0.525$, $T = 0.15$ with particles detected as P3 particles colored in red. Inset: target and threshold for P3 structure detection. In the middle: simulation snapshots at $\phi = 0.475$, $T = 0.15$ with particles detected as Z1 colored in green. Inset: target and threshold for Z1 structure detection. On the right: simulation snapshots at $\phi = 0.225$, $T = 0.12$ with particles detected as S colored in blue. Inset: target and threshold for S structure detection.

of the S crystal in the dmo-as1 (in Figure 3 on the right) a caveat must be added: the Z1 lattice and the S lattice are the only identified lattices with the same bonding pattern – two non-parallel bonds and one parallel bond. In this exceptional case, we distinguish the S-particles from the Z1-particles by additionally checking if the particle is bonded within a non-parallel loop of size six (6-np loop). Once the bonding patterns have been determined, the crystal structure detection works analogously, where the target structure is that all three (3/3) bonded neighbours are bonded within a 6-np loop, while the threshold is that at least one of the neighbours is bonded in a 6-np loop (1/3).

The detected crystal structures for the dmo-s1 systems – P4, Z2 and Z1 – in Figure 4 further confirm that the crystal identification via bonding patterns is very accurate and rather strict, as can be seen especially well in the fully bonded P4 clusters, where all particles at the boundary of the cluster are excluded due to the fact that they are not fully bonded. For the Z2 system (Figure 4 in the center) we can observe that the defect rows are also excluded form the Z2 crystallite. We note that the defect lines themselves adhere to an Z1-bonding pattern and are detected as such by the algorithm, as illustrated – albeit by a

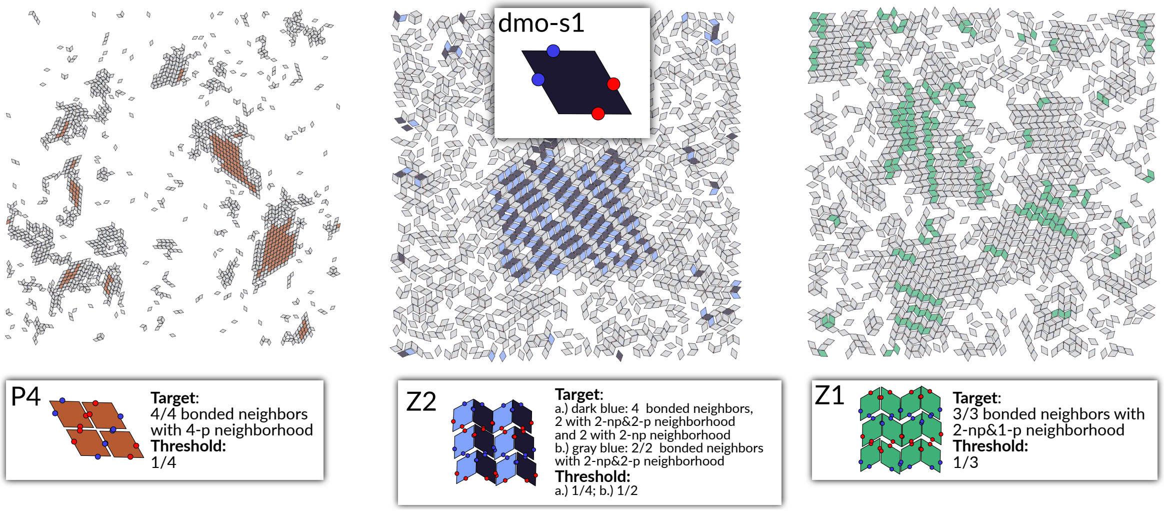

FIG. 4. **Crystal structure detection for dmo-s1.** On the left: simulation snapshots at $\phi = 0.15$, $T = 0.11$ with particles detected as P4 particles colored in brown. Inset: target and threshold for P4 structure detection. In the middle: simulation snapshots at $\phi = 0.5$, $T = 0.16$ with particles detected as Z2 colored in black (2-np-2p bonding pattern) and light blue (2-np bonding pattern). Inset: target and threshold for Z2 structure detection. On the right: simulation snapshots at $\phi = 0.5$, $T = 0.14$ with particles detected as Z1 colored in green. Inset: target and threshold for Z1 structure detection.

different snapshot – in Figure 4 on the right hand side. In dmo-s1, Z1-crystalline rows are in fact most often seen as defect lines within Z2, and it might be interesting to investigate in the future if the Z1-crystal for dmo-s1 only grows as a defect of Z2. At this point, we note and reiterate, that it is due to the chosen low crystallinity threshold, that we can distinguish the crystal symmetry of a single defect row and thus reason about it. Finally, we show the detection of the crystal polymorph of dmo-s2 in Figure 5: P3, Z3 and PZ. While P3 in Figure 5 on the left , and to a lesser extent PZ in Figure 5 on the right, exhibit clearly detectable crystallites, there are many small scattered Z3 environments without a clear crystal symmetry visible, highlighting the importance of maintaining minimum threshold of – in this case five – bonded crystalline particles.

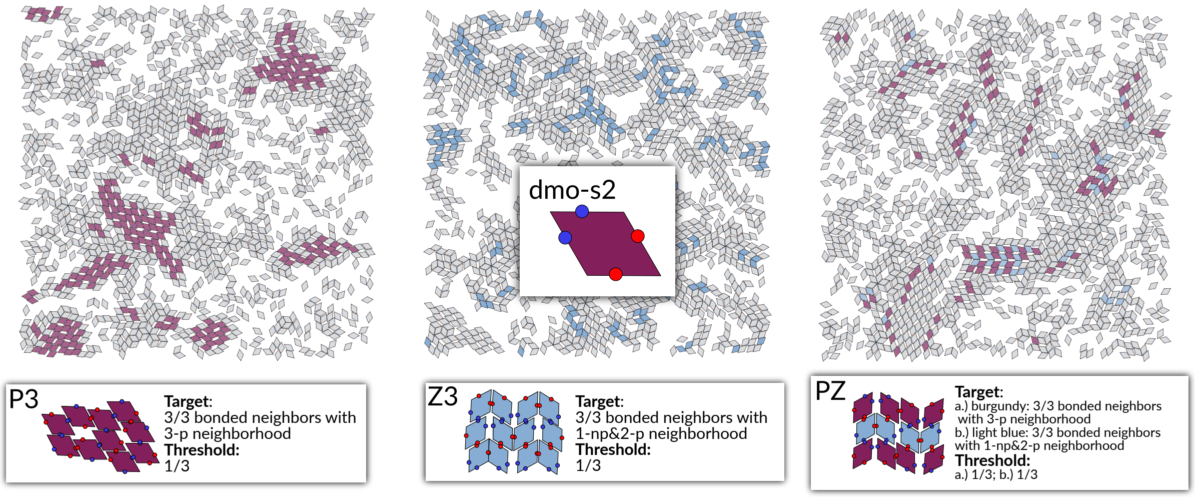

FIG. 5. **Crystal structure detection for dmo-s1.** On the left: simulation snapshots at $\phi = 0.475$, $T = 0.14$ with particles detected as P3 particles colored in red. Inset: target and threshold for P3 structure detection. In the middle: simulation snapshots at $\phi = 0.5$, $T = 0.13$ with particles detected as Z3 colored in blue. Inset: target and threshold for Z3 structure detection. On the right: simulation snapshots at $\phi = 0.5$, $T = 0.14$ with particles detected as PZ colored in red (3-p bonding pattern) and blue (1-np-2p bonding pattern). Inset: target and threshold for PZ structure detection.

II. LONGER RUNS FOR DMO-AS1

To further investigate whether crystal growth is fundamentally limited or rather very slow, we extended the simulation time from approximately 2.5×10^7 MC-sweeps to 3.5×10^7 MC-sweeps within the T/ϕ region of interest for the dmo-as1 systems. As discussed in the main text, we observe that at the highest packing fractions, the overall crystallinity increases from 0.25 to beyond 0.3, reaching as high as 0.5 in some cases. In contrast, at lower packing fractions, the overall crystallinity remains unchanged. Figures 6 and 7 illustrate these statistical findings by comparing crystallite structures at shorter and longer simulation times for two distinct packing fractions. For the higher packing fraction ($\phi = 0.5$), significant growth of the P3 crystallite is evident when comparing the state at 2.5×10^7 MC-sweeps (Figure 6a) to that at 3.5×10^7 MC-sweeps (Figure 6b). Conversely, at a lower packing fraction ($\phi = 0.35$), no substantial growth is observed in the P3 and Z1 crystallites, as shown by comparing the shorter simulation time (Figure 7a) with the longer simulation

time (Figure 7b). It is worth noting that even for the higher packing fraction, the noticeable crystallite growth occurs over a prolonged timescale of 1×10^7 MC-sweeps, which strikes us as unusually long for patchy systems of this type.

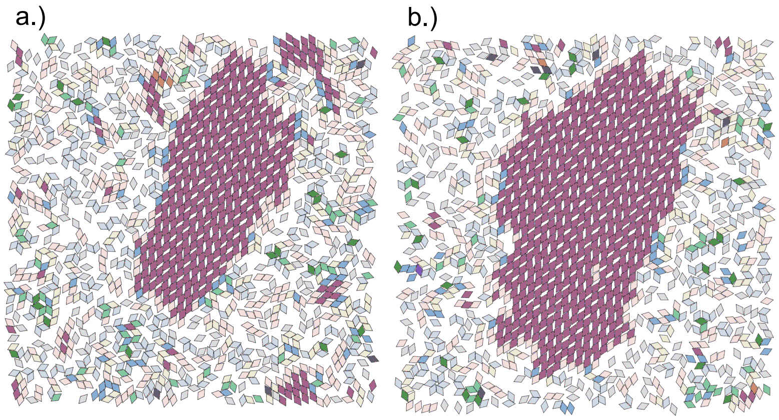

FIG. 6. Snapshot comparison of the same system for dmo-as1, for $\phi = 0.525$, $T = 0.16$ at a.) MC sweeps$\approx 2.5 \times 10^7$ b.) MC sweeps$\approx 3.5 \times 10^7$.

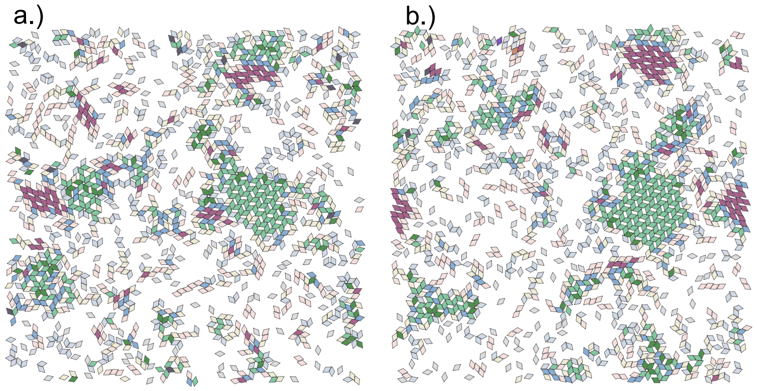

FIG. 7. Snapshot comparison of the same system for dmo-as1, for $\phi = 0.35$, $T = 0.12$ at a.) MC sweeps$\approx 2.5 \times 10^7$ b.) MC sweeps$\approx 3.5 \times 10^7$.

III. RUNS FOR LARGER SYSTEM SIZES FOR DMO-AS1 AT N=3000

Additionally we investigate the effect of system size on the assembly and simulate systems with 3000 particles for three selected state points: $\phi = 0.225, T = 0.12$, $\phi = 0.4, T = 0.14$ and for $\phi = 0.475, T = 0.15$. For each state point we conduct three parallel runs for a total simulation time 1.6×10^7 MC sweeps. We find the crystallinity to be slightly lower in the larger system compared to the smaller, but within the error bars of the standard deviation. Note that instead of 16 parallel runs, we only use three parallel runs, which might explain the deviation. We summarize the overall crystallinity of these three state points in Table 1. To illustrate these findings we show two snapshots for two different parallel runs for

ϕ	T	crystallinity N=1500	crystallinity N=3000
0.225	0.12	0.132 ± 0.049	0.114 ± 0.033
0.4	0.14	0.131 ± 0.053	0.125 ± 0.027
0.475	0.15	0.170 ± 0.043	0.135 ± 0.042

TABLE I. Comparison of overall crystallinity for smaller systems (number of particles N=1500) and larger systems (N=3000) at $\approx 1.6 \times 10^7$ MC-sweeps for three different state points.

$\phi = 0.475$ and $T = 0.15$ in Figure 8, where Figure 8a shows a configuration with more P3 crystallites and Figure 8b depicts a configuration with more Z1.

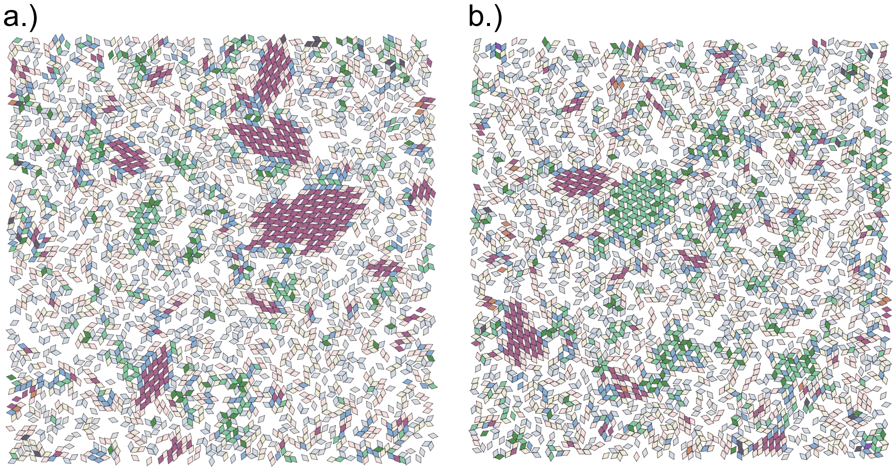

FIG. 8. Snapshots from two different parallel runs of a large-scale dmo-as1 system: $N = 3000$, $\phi = 0.475$ and $T = 0.15$; a.) more P3, b.) more Z1.

[1] P. J. Steinhardt, D. R. Nelson, and M. Ronchetti, Bond-orientational order in liquids and glasses, Physical Review B **28**, 784 (1983).
